# Association of Comorbidities with Adverse Outcomes in Adults Hospitalized with Respiratory Syncytial Virus (RSV) Infection: A Retrospective Cohort Study from Switzerland (2022–2024)

**DOI:** 10.3390/v17081030

**Published:** 2025-07-23

**Authors:** Neetha Joseph, Elisa D. Bally-von Passavant, Giorgia Lüthi-Corridori, Fabienne Jaun, Sandra Mitrovic, Jörg Daniel Leuppi, Maria Boesing

**Affiliations:** 1 University Institute of Internal Medicine, Cantonal Hospital Baselland, CH-4410 Liestal, Switzerland; 2 Faculty of Medicine, University of Basel, CH-4056 Basel, Switzerland; 3 Center for Executive and Continuing Education, Harvard T.H. Chan School of Public Health, Boston, MA 02115, USA; 4 Central Laboratory, Cantonal Hospital Baselland, CH-4410 Liestal, Switzerland

**Keywords:** respiratory syncytial virus, comorbidities, severe course

## Abstract

Introduction: Respiratory Syncytial Virus (RSV) infection causes seasonal respiratory illness in both children and adults, with increasing recognition of its impact in older adults with chronic comorbidities. This study aimed to characterize adult patients hospitalized with RSV infection in Switzerland and identify comorbidities linked to poor outcomes. Methods: Adults hospitalized with RSV infection between May 2022 and April 2024 at a Swiss public teaching hospital were included in this retrospective observational study. To assess the association between comorbidities and patient outcomes, separate multivariable regression analyses for each comorbidity, adjusted for age and sex, were performed. The primary composite endpoint was ’severe course’ (in-hospital death or intensive care unit (ICU) admission), secondary endpoints included in-hospital death, ICU admission, and length of stay. Results: Among 136 included patients (mean age 78, 38% male), 98% had comorbidities, most commonly cardiovascular (75.7%), respiratory (51%), and chronic kidney disease (CKD) (36.7%). Further, 18.4% experienced a severe course. The ICU admission rate was 14.0%, in-hospital mortality 6.6%, and the median hospital stay of survivors was 6 days (IQR 4–10). CKD was significantly associated with severe course (OR 2.64, *p* = 0.045) and in-hospital mortality (OR 11.6, *p* = 0.025), while immunosuppression predicted ICU admission (OR 5.7, *p* = 0.018). Length of stay was not linked to any comorbidities. Conclusions: In this cohort of hospitalized adults, mainly elderly individuals with chronic comorbidities were tested positive for RSV. CKD and immunosuppression were associated with severe course. Prevention strategies, including RSV vaccination, should prioritize these high-risk populations.

## 1. Introduction

The human Respiratory Syncytial Virus (RSV) is a prevalent respiratory virus belonging to the *Pneumoviridae* family, which shows distinct seasonal patterns, typically peaking during winter seasons depending on geographical location and climate [1,2,3]. RSV transmission occurs through contact with respiratory droplets from infected individuals or contaminated surfaces via large droplet inoculation in the eyes, nose, or mouth, requiring close contact with an RSV-infected subject or autoinoculation to the face [4]. Although RSV is known to be a leading cause of respiratory tract infections, particularly in children [5], recent data reveal that RSV infections are also common among adults, leading to severe respiratory infections [6,7]. According to a multicentric multinational study conducted across 15 countries in North America, Europe, and East Asia, RSV was the third most common respiratory virus causing moderate-to-severe influenza-like illness episodes in adults aged 65 and older [8]. In this population, RSV can lead to complications requiring emergency care or hospitalization [7,9]. Unlike the pediatric population, where disease severity is largely driven by immune immaturity, in older adults, immunosenescence and comorbid conditions likely contribute to heightened vulnerability and more severe clinical outcomes [8,9,10].

Despite growing awareness, the burdens of RSV among adults remain under-recognized [10]. Comorbidities, such as chronic obstructive pulmonary disease (COPD), congestive heart failure (CHF), chronic kidney disease (CKD), pre-existing cardiopulmonary conditions, and obesity, have been consistently associated with increased severity and worse functional outcomes in RSV-infected adults [11,12,13]. Moreover, immunosuppression, whether due to organ transplantation, chemotherapy, or chronic corticosteroid use, has also been linked to severe RSV cases [14,15,16]. In addition, hospitalization due to RSV infection has been found to be associated with acute cardiac events, especially in patients with cardiovascular risk [11,12,17]. This leads to a high risk of requiring costly treatments, higher hospitalization rates, and mortality [18]. To address this, the Centers for Disease Control and Prevention (CDC) have published a comprehensive list of conditions that increase the risk of a severe RSV course, providing guidance for clinicians on identifying high-risk individuals [19].

In response to this growing public health concern, preventive strategies such as vaccination have recently become available. In 2023, the first RSV vaccines Arexvy^®^ and Abrysvo^®^) were approved for individuals aged 60 and older in the USA and European Union, and both received Swiss regulatory approval in 2024 [20,21]. Another vaccine (mResvia) is currently under review [21].

However, to effectively guide the implementation of such measures, a clearer understanding of the adult populations most at risk of severe outcomes of RSV infection is required. This study aimed to describe the clinical and comorbidity profiles of adult patients hospitalized with RSV infection in a Swiss public teaching hospital. By providing recent, real-world data from Switzerland, this study seeks to identify comorbidities associated with poor outcomes in this specific and similar European healthcare settings. Improved characterization of high-risk patients may inform clinical decision making and support vaccination strategies in vulnerable adult populations.

## 2. Materials and Methods

### 2.1. Design and Setting

In this retrospective, single-center observational study, we analyzed clinical routine data from all adult patients who were admitted to the Cantonal hospital Baselland (Kantonsspital Baselland, KSBL) with RSV infection, confirmed by polymerase chain reaction (PCR), between May 2022 and April 2024.

KSBL is a district general teaching hospital covering a stable population of 280,000 in northwest Switzerland [22]. Patients admitted to one of the two sites in Liestal and Bruderholz were included.

### 2.2. Inclusion and Exclusion Criteria

Adult patients (18 years or older) who were hospitalized for at least one night at KSBL with a laboratory-confirmed RSV infection (detected by PCR using the GeneXpert^®^ system, Cepheid, Sunnyvale, CA, USA) were eligible for inclusion in this study. In particular, patients hospitalized for reasons other than respiratory symptoms were also included. This approach aimed to capture the broader impact of RSV, particularly its potential to exacerbate chronic comorbidities, such as COPD and heart failure, which may in turn contribute to hospitalization. At KSBL, patients with respiratory symptoms likely to be of infectious origin were generously tested for RSV. Patients who denied consent for their clinical routine data to be analyzed (general research consent) were excluded from the study.

### 2.3. Data Collection

RSV was identified from respiratory swabs by multiplex PCR. Basic demographic and hospitalization data, including sex, age, and length of hospital stay (LOHS), were extracted from the hospital’s electronic database. Additional clinical data were manually collected from patients’ electronic records, including vital signs upon admission (body temperature, heart rate, blood pressure, respiratory rate, peripheral oxygen saturation (SpO_2_), chronic comorbidities, and smoking history). Moreover, clinical manifestations upon admission, laboratory data (including white blood cell count, neutrophil percentage, lymphocyte count, C-reactive protein (CRP), serum creatinine, estimated glomerular filtration rate (e-GFR)), oxygen supplementation, and outcome (in-hospital death, admission to intensive care unit, mechanical ventilation, and intubation days) were collected.

The assessed comorbidities included coronary heart disease, CHF, peripheral vascular disease (PVD), previous cerebrovascular insult (CVI) or transient ischemic attack (TIA), arrhythmia, COPD, asthma, diabetes, CKD, immunosuppression, chronic liver disease (CLD), and leukemia or lymphoma. ‘Immunosuppression’ was defined as the presence of any of the following conditions or treatments: HIV infection or AIDS, history of organ transplantation, chemotherapy, prolonged corticosteroid use, or immunomodulating medications such as methotrexate or biologics. Comorbidities were identified from discharge reports and other clinical documentation available at the time of hospitalization. A comorbidity was considered present if explicitly documented, and absent if not mentioned. Missing data in the patients’ admission status, smoking status, and laboratory values were reported in the descriptive analyses. As there were no missing values for comorbidities, age, or sex, no imputation methods were required for the further statistical analyses.

The collected data were stored and managed using Research Electronic Data Capture (REDCap^®^, Vanderbilt University, Nashville, TN, USA), a secure, web-based software platform designed for clinical research data management [23,24].

### 2.4. Outcomes

The primary outcome was a severe course, defined as the composite of intensive care unit (ICU) admission or in-hospital death. When aiming to predict a severe course of disease, it is important to take into account that the admission to ICU is always an individual decision that is not only dependent on objective indication, but also on patient preferences, family decisions, and contextual factors. Thus, when ICU admission is clinically indicated, individual patients’ beliefs and circumstances play an important role in the respective outcome. Consequently, ‘in-hospital death’ and ‘ICU’ should not be separated in terms of severe course because both could indicate the same level of severity.

As secondary endpoints, ICU admission and in-hospital death were assessed separately, as well as LOHS.

### 2.5. Statistical Analysis

The descriptive statistical analyses were performed using REDCap^®^, version 13.8.1. Categorical variables are presented as absolute and relative frequencies. Continuous variables are presented as median and interquartile range (IQR). To assess the association between comorbidities and patient outcomes, we performed separate multivariable regression analyses for each comorbidity listed in Section 2.4, adjusting for age and sex. For each comorbidity, a multivariable regression was conducted with the outcome of interest as the dependent variable, and the respective comorbidity, and age and sex as independent variables. Logistic regression was used for the dichotomous outcomes ‘severe course’, ‘ICU admission’, and ‘in-hospital death’. Zero-truncated negative binomial regression was used for the continuous outcome ‘LOHS’.

### 2.6. Ethical Considerations

This study was approved by the ethics committee of Northwestern and Central Switzerland (ENKZ, BASEC Project-ID 2021-00964) on 10 June 2021. Patients who objected to the use of their clinical routine data for research purposes (general research consent) were excluded from the study.

## 3. Results

### 3.1. Patient Characteristics

Between May 2022 and April 2024, 1846 adult patients hospitalized at KSBL were tested for infection with RSV (multiplex PCR), out of which 153 obtained a positive result. Further, 17 patients were excluded due to a denied general research consent, thus data from 136 patients were analyzed.

The characteristics of the included patients are detailed in Table 1. The median age at hospital admission was 79.5 years, with the youngest being 38 years and the oldest 99 years old. Additionally, 61.8% of the patients were female. Among the patients with available smoking status (*n* = 37, 27.2%), 89.1% were current or former smokers. Most patients (77.2%) were admitted due to respiratory symptoms, while others (*n* = 31, 22%) were admitted for other reasons, including cardiovascular (2.2%), gastroenterological (5.9%), and orthopedic diagnoses (3.7%).

Vital signs measured at admission revealed that patients were mostly normocard (median heart rate 89 bpm, IQR 77.8–118.5). The average systolic blood pressure was slightly hypertonic at 142 mmHg (IQR 120–150). Within our study group, 41 patients (30.4%) presented with fever (body temperature > 38 °C) upon admission. The median peripheral oxygen saturation levels were 93% (IQR 90–95), including values of 27 patients (19.8%) whose measurements were taken under supplementary nasal oxygen. Among 128 patients for whom a respiratory rate was documented, 62 (48.1%) presented normopneic with rates below 20 per minute, while 65 (50.7%) presented with tachypnea.

The majority of patients presented with an increased CRP (median 44 mg/L, IQR (17.75–118.5) and increased neutrophil–lymphocyte ratio (NLR, median 7.51, IQR (3.96–14)), indicating inflammation, while white blood cell count average was within the normal range.

### 3.2. Comorbidities

Almost all patients (134 out of 136, 98.5%) had one or more chronic underlying disease (Table 2). The most frequent type were chronic cardiovascular comorbidities, as present in over three quarters of the patients (75.7%). Moreover, 79 (58.0%) patients had arterial hypertension and 33 (24.2%) coronary artery disease. The most common non-cardiovascular comorbidities were CKD (36.7%), followed by diabetes mellitus (19.8%) and COPD (18.3%). Other comorbidities included stroke or TIA (*n* = 24/136, 17.6% neurological disorders (*n* = 27/136, 19.8%), chronic hematological disease (*n* = 13/136, 9.5%), solid tumor (n = 15/136, 11.2%), asthma (n = 16, 11.7%), and immunosuppression (*n* = 11/136, 8.0%). Further, 17 patients (30.2%) presented with a BMI > 30 kg/m^2^.

### 3.3. Outcomes of Hospitalized Patients with RSV

Patient outcomes, overall and grouped by comorbidities, are presented in Table 3. Overall, 25 patients (18.4%) had a severe course (ICU admission or in-hospital death); 19 patients (14%) were admitted to the ICU during their stay, with 11 (8.0%) requiring some type of mechanical ventilation, either invasive (*n* = 4, 2.9%) or non-invasive (*n* = 8, 5.8%). Further, 3 patients received high-flow oxygen therapy. Nine patients (6.6%) died during hospitalization. The overall median length of hospital stay was six days (IQR: 4–10).

#### 3.3.1. Primary Outcome: Severe Course

The highest rates of severe course (ICU admission or in-hospital death) occurred in patients with immunosuppression (*n* = 4, 36.4%), followed by coronary artery disease/myocardial infarction (*n* = 10, 27.8%), CKD (*n* = 13, 26%), and CLD (*n* = 3, 25%). Out of the four patients with immunosuppression who experienced a severe course, one was undergoing chemotherapy, one was receiving prolonged corticosteroid therapy, and two were undergoing prolonged corticosteroid therapy combined with immunomodulating therapy (methotrexate and nintedanib, respectively).

**Figure 1:**
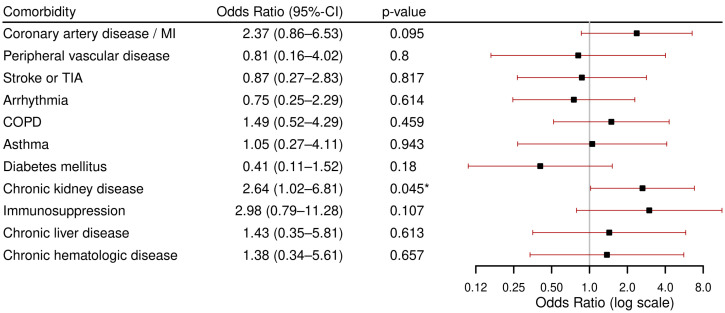
Results of multivariable logistic regression models for severe course (ICU admission or in-hospital mortality) per comorbidity, each adjusted for age and sex. Abbreviations: ICU: Intensive Care Unit, MI: Myocardial Infarction, TIA: Transient Ischemic Attack, COPD: Chronic Obstructive Pulmonary Disease. * *p* < 0.05.

The results of the regression analyses for comorbidities associated with severe course are displayed in Figure 1. CKD was found to be the only comorbidity that was significantly associated with severe course, with an odds ratio (OR) of 2.64 (95%CI: 1.02–6.81, *p* = 0.045).

#### 3.3.2. Secondary Outcomes: ICU Admission, in-Hospital Death, and LOHS

**Figure 2:**
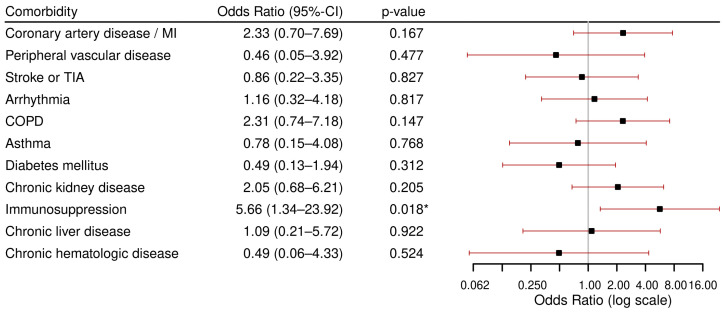
Results of multiviariable logistic regression models for ICU admission per comorbidity, each adjusted for age and sex. Abbreviations: ICU: Intensive Care Unit, MI: Myocardial Infarction, TIA: Transient Ischemic Attack, COPD: Chronic Obstructive Pulmonary Disease. * *p* < 0.05.

The highest rate of ICU admissions was observed among the 11 patients with immunosuppression; 4 (36.4%) were admitted to the ICU. Patients with COPD also showed a high ICU admission rate at 24.0% (*n* = 6). The result of the regression analyses for each comorbidity modeling the outcome ICU admission are reported in Figure 2. The analysis revealed a statistically significant association between immunosuppression and ICU admission, with an odds ratio (OR) of 5.7 (95%CI: 1.3–23.9, *p* = 0.018). No other comorbidity was found to be associated with ICU admission.

**Figure 3:**
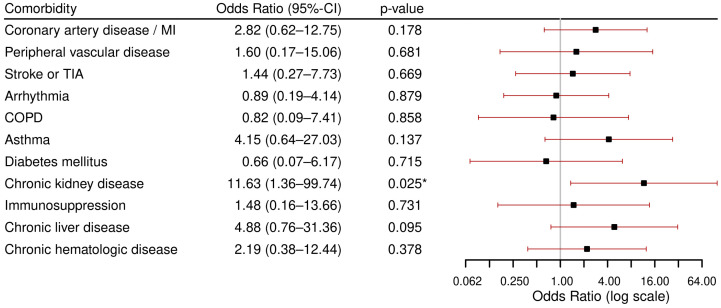
Results of logistic multivariable regression models for in-hospital death per comorbidity, each adjusted for age and sex. Abbreviations: MI: Myocardial Infarction, TIA: Transient Ischemic Attack, COPD: Chronic Obstructive Pulmonary Disease. * *p* < 0.05.

The highest in-hospital mortality was observed in the group of patients with CLD (*n* = 11, 16.7%) and in the group of patients with CKD (*n* = 8, 16%), followed by patients with chronic hematologic disease (*n* = 2, 15.4%) and coronary artery disease/myocardial infarction (*n* = 5, 13.9%). The results of the regression analyses performed to assess the association between comorbidities and in-hospital death are displayed in Figure 3. The analyses revealed an association between CKD and in-hospital death, with an OR of 11.6 (95%CI (1.36–99.74), *p* = 0.02). No other comorbidity was found to be associated with in-hospital death.

**Figure 4:**
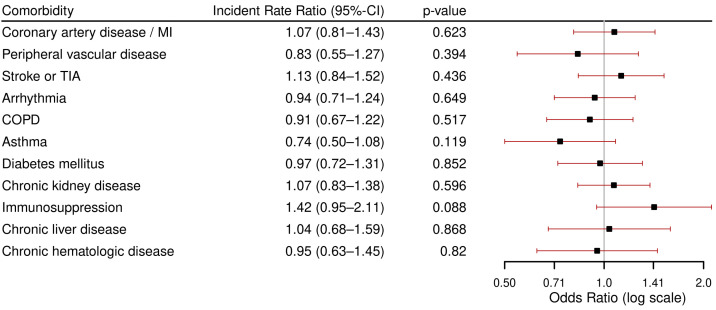
Results of multivariable zero-truncated negative binomial regression analysis for length of hospital stay in survivors (*n* = 127) per comorbidity, each adjusted for age and sex. Abbreviations: MI: myocardial infarction, TIA: Transient Ischemic Attack, COPD: Chronic Obstructive Pulmonary Disease.

Patients with immunosuppression also had the longest median hospital stays, with a duration of 9 days, followed by patients with previous stroke or TIA, CLD, and coronary artery disease/myocardial infarction, all with a median duration of 8 days. The results of the regression analyses for LOHS are presented as incident rate ratios (IRR) in Figure 4. Patients with asthma had the lowest IRR (IRR 0.74), indicating a shorter LOHS than the average patient, while patients with immunosuppression had the highest IRR (IRR 1.42). However, none of the comorbidities were significantly associated with LOHS.

## 4. Discussion

This retrospective observational cohort study analyzed clinical characteristics, comorbidities and their association with outcomes of adult hospitalized patients with RSV over a two year period, aiming to identify populations at risk for severe disease course. The main findings are as follows:

- Patients who tested positively for RSV infection were mainly elderly individuals and nearly all had chronic underlying comorbidities, mainly cardiovascular, respiratory diseases and CKD.
- The highest rates of severe course (ICU admission or in-hospital death) were found in patients with immunosuppression, followed by coronary artery disease/myocardial infarction, chronic hematological disease, CKD and CLD.
- CKD was associated with both severe course and in-hospital mortality.
- Immunosuppression was associated with ICU admission.

### 4.1. Patient Characteristics

Our cohort had a median age of almost 80 years and a predominance of female patients (61.8%). These demographics are consistent with previous studies investigating RSV-related hospitalizations in older adults. Osei et al. reported that 92% of RSV-related hospitalizations in the European Union occurred among individuals aged 65 years and older. Similarly, Havers et al. found that RSV was associated with a significant burden of hospitalizations, ICU admissions, and in-hospital deaths among adults, with the highest rates observed in those aged ≥ 75 years (median age 75 years; 58.2% female) [25,26]. These findings highlight the disproportionate impact of RSV on older populations. Urchueguía-Fornes et al. also demonstrated an age-related increase in RSV hospitalization rates, with the highest in individuals aged ≥ 80 years [27]. The results of the present study are consistent with those of other studies, confirming that RSV predominantly affects older adults, as reflected by a high median age (79.8 years) of hospitalized patients. Additional clinical characteristics, such as radiological findings and blood gas measurements at admission, should be considered in a full characterization of patients’ presentation.

### 4.2. Comorbidities

Our data demonstrate that 98.5% of hospitalized RSV-infected patients had chronic underlying conditions. Cardiovascular comorbidities represent the most common underlying comorbidity leading to hospitalization among our study population, followed by chronic lung diseases and CKD, highlighting significant burden in adults with chronic underlying conditions. Moreover, among cardiovascular comorbidities, arterial hypertension was found to be the most prevalent. Our results are in line with previous findings [7,12,28,29,30]. A recently published Danish–Scottish study revealed that certain levels of variation were identified among the comorbidities that resulted in hospitalization of patients with RSV [31]. The study found that in Denmark, patients with CKD exhibited the highest rate of hospitalization (19.4 per 1000), while patients with asthma demonstrated the lowest rate (3.1 per 1000). In contrast, in Scotland, patients with COPD exhibited the highest rate of hospitalization (9 per 1000), while patients with chronic liver disease exhibited the lowest rate (2.1 per 1000). Therefore, it can be hypothesized that there is a certain level of variation in comorbidities between countries, which is potentially attributable to differences in healthcare systems, diagnostic practices, or data collection methods. The primary reason for hospital admission was respiratory symptoms, as reported in 77.2% of the patients. Tachypnoea was a common clinical feature, observed in approximately half of the patients, consistent with findings from previous studies [32,33]. On the other hand, 31 patients (22.8%) were hospitalized for non-respiratory reasons. Even though RSV may have been an incidental finding or due to nosocomial infection in some of these cases, we can hypothesize that non-respiratory underlying diseases decompensated (e.g., CHF) or led to complications (e.g., falls) in the presence of an RSV infection. Indeed, previous studies have demonstrated that RSV infection can exacerbate non-respiratory chronic conditions, such as CHF, diabetes and CKD, leading to increased hospitalization rates and severe complications [17,30,31].

### 4.3. Outcomes of Hospitalized Patients with RSV

In our study, 14% of patients hospitalized with RSV infection required ICU admission, a rate comparable to those reported in recent studies by Volling et al. and Goldman et al. (15% and 16%, respectively) [29,34]. Several studies have highlighted the severity of RSV infections in adults, especially in those aged above 60 years with the necessity of ICU admission [17,35,36]. The mortality rate associated with RSV infection in our study at 6.6% is consistent with findings from previous studies [36]. When comparing RSV and influenza, previous studies reported higher mortality in RSV [37]. These findings highlight the significant mortality risk associated with RSV in hospitalized adults, particularly those with underlying chronic disease [31,36]. Our data show that patients with chronic hematological diseases, immunosuppression, chronic liver disease and CKD exhibited a severe course of illness, with the highest rate of mortality in chronic hematological patients, as previously reported [15,17,28,38,39]. Due to the small sample size and the heterogeneity of patients with immunosuppression, no further conclusions can be drawn with regard to which type of immunosuppression may pose a particular risk for a severe disease course. Further studies are needed to investigate this association in this vulnerable patient group.

Factors such as underlying comorbidities, age, and immune status are often cited as key contributors to poor outcomes in viral respiratory infections [36,40,41,42]. In our study, CKD emerged as a significant predictor of severe course and in-hospital death in the regression models, indicating that CKD may play an important role in influencing the prognosis. This underscores the need for closer monitoring and individual management strategies in this population. Earlier studies reported increased hospitalization rates, respiratory failure, prolonged hospital stays and high mortality in CKD patients [30,43]. Alternative factors such as viral load, better access to healthcare systems, and socioeconomic factors could also have influenced the severity of disease progression, implicating the necessity for further investigation into what other variables could be further predictors of poor outcomes in RSV patients.

It is evident that pediatric immunocompromised patients with RSV often require hospitalization, which in turn directly or indirectly leads to a relevant delay in the initiation of necessary treatment for the underlying diseases or the modification of existing therapy [44]. A recent study from the university hospitals of Lausanne and Geneva in Switzerland concluded that among pediatric and adult immunocompromised participants, the patients admitted to ICU or the deceased were mostly adults [39]. Accordingly, our regression analysis also showed that immunosuppressed patients have an increased risk of ICU admission. We therefore conclude that special screening procedures for RSV should be established in this patient population, which would subsequently lead to special preventive strategies and an awareness of severe cases being established, especially among primary care providers, e.g., in general practices and emergency departments.

Consistent with findings from other studies [30,31], our results indicate that CKD is highly prevalent among patients hospitalized with RSV. Moreover, in our analysis, CKD specifically emerged as a significant predictor of in-hospital death. Celante et al. identified several factors associated with in-hospital mortality alongside CKD, including age over 85 years, acute respiratory failure, invasive mechanical ventilation, and neutropenia [36]. Further research is required to better characterize population-specific risk factors and improve prevention and treatment strategies.

In our study, patients had a median LOHS of 6 days (IQR: 4–10), which is consistent with findings from a retrospective Canadian study by Vollig et al., in which the reported median LOHS was also 6 days [29]. Similarly, a multi-center retrospective chart review from the USA found a median LOHS of 6.0 days (IQR: 3.0–9.0) for adults with RSV, which identified age on admission, smoking status, Charlson Weighted Index of Comorbidity (CWIC) and presence of pneumonia as predictors of prolonged LOHS [45], highlighting the need for enhanced clinical management and resource planning for high-risk groups. Furthermore, many studies have consistently shown that RSV infection in adults results in longer hospital stays compared to influenza [37,41,46]. For instance, older adults and those with chronic conditions tend to have longer hospital stays [43,47]. In our study, we could not identify any predictors for prolonged LOHS among the assessed comorbidities. However, patients with Stroke/TIA and immunosuppression had the longest hospital stay in our cohort.

Since vaccines for RSV are now available, we assume that certain comorbidity groups (especially elderly individuals, individuals with CKD, coronary artery disease, immunocompromised patients) will benefit from early seasonal vaccination, so that the hospitalization rate and the number of adverse outcomes among these groups can be reduced. Further comprehensive and larger studies are essential to enable a more thorough evaluation of comorbidity groups, thereby facilitating the development of a more evidence-based and generalized recommendation for vaccination strategies. These studies should focus on a diverse patient population, considering variables such as age, underlying health conditions, and regional factors, to provide a clearer understanding of which groups would benefit most from targeted vaccination. Additionally, long-term follow-up data will be crucial in assessing the sustained efficacy of the vaccination and its impact on hospitalization rates and disease outcomes across different comorbidity groups. Moreover, we suggest that a detailed analysis of RSV subtypes could provide more precise insights into the distinct risks of severe illness linked to each subtype. This study did not consider the potential for co-infections with other respiratory viruses. It is possible that such co-infections may have contributed to the observed severity of the disease. Stratifications regarding sub-type and analyses of co-infections may improve the accuracy of risk assessments and facilitate the development of more targeted prevention strategies, which in turn enables a more effective cost–benefit evaluation.

### 4.4. Limitations

There are several limitations to this study that must be acknowledged. First, as a retrospective study, the quality of the data is inherently dependent on the accuracy and completeness of documentation in the patient files. This reliance on historical records means that any discrepancies, errors, or omissions in the data could have an impact on the findings. In particular, incomplete documentation may have resulted in missing information, especially with respect to reported comorbidities and key clinical variables. This could lead to biases or inaccurate conclusions, particularly if vital clinical data were underreported or not properly recorded. Furthermore, the reliance on discharge letters and routine clinical documentation to identify comorbidities may have introduced misclassification bias, as these sources can include preliminary or unverified diagnoses that do not always reflect confirmed or chronic conditions.

Second, RSV testing was not performed systematically for all hospitalized patients. Testing was primarily conducted in individuals presenting with respiratory symptoms or signs of infection, which may have led to underdiagnoses in asymptomatic or atypical cases. As a result, there is a risk of selection bias, as the cohort may over-represent patients with more pronounced clinical presentations. This limitation reduces the generalizability of our findings.

Third, the sample size of hospitalized RSV patients in this study was relatively small. This limitation directly affected the number of events related to our primary endpoint, as fewer events could mean less statistical power to detect significant associations. With a smaller number of cases, the ability to adjust for confounding factors such as age and sex becomes more limited. In turn, this could influence the precision of the estimates and the generalizability of the findings. A larger cohort might have allowed for more robust, multivariable statistical analyses and a better understanding of the interplay between the factors contributing to adverse outcomes in RSV patients.

Regarding the choice of outcomes, it is important to recognize that ICU admission and LOHS can be influenced by institutional policies, clinical judgment, and individual patient preferences, and therefore may not exclusively reflect disease severity. These potential sources of bias should be taken into account when interpreting the results. Nonetheless, the combination of chosen outcomes provides a broader perspective on disease severity by capturing different clinical dimensions. The use of validated severity measures, such as the Pneumonia Severity Index (PSI), as primary or additional secondary outcomes could provide a more nuanced assessment of disease severity and should be considered in future prospective studies.

Lastly, our study lacks a control cohort, which limits the ability to directly compare RSV-infected patients with non-infected individuals. This limitation arises from the retrospective design, as the study was not structured as a controlled investigation. Future prospective studies should incorporate appropriately matched control groups to enable more definitive assessments of the impact of RSV infection and its associated comorbidities.

Despite these challenges, it is important to note that the individual risk for severe outcomes in RSV patients may still be higher than anticipated, even with a limited sample size. This could suggest that, although the data may be constrained, there is a real and significant risk of adverse outcomes for those affected by RSV, which warrants further investigation in larger, prospective studies to confirm these findings and explore the potential impact of confounding variables more thoroughly.

## 5. Conclusions

In this Swiss cohort of hospitalized adults, mainly elderly individuals tested positive for RSV. The majority were affected with chronic comorbidities such as CKD, cardiovascular diseases and immunosuppressive conditions. CKD and immunosuppression were associated with severe outcomes. Preventive strategies such as RSV vaccination should be prioritized in these high-risk groups to reduce the burden of severe infections and hospitalization, ultimately improving patient outcomes and reducing healthcare costs. Larger, more comprehensive, and prospective studies are essential to refine our unbiased understanding of the risk factors that predict severe outcomes of RSV infection.

## Figures and Tables

**Table 1:** Baseline characteristics.

	All (*n* = 136)	Missing *n* (%)
Demographic		
Age at admission (years), median (IQR)	79.50 (72–87)	
Female *n* (%)	84 (61.8%)	
Smoking Status		99 (72.8%)
Former Smoker, *n* (%)	19 (51.4%)	
Current smoker, *n* (%)	14 (37.8%)	
Never smoked, *n* (%)	4 (10.8%)	
Reason for hospital admission		
Respiratory, *n* (%)	105 (77.2%)	
Cardiovascular, *n* (%)	3 (2.2%)	
Gastroenterogical, *n* (%)	8 (5.9%)	
Orthopedic, *n* (%)	5 (3.7%)	
Other, *n* (%)	15 (11.0%)	
Vital Signs at admission		
Respiratory rate, median, brpm (IQR)	21 (18–25)	8 (5.8%)
SpO_2_ in %, median (IQR)	93 (90–95)	
Supplemental oxygen, *n* (%)	26 (19.1%)	
Fever ^1^, *n* (%)	41 (30.4%)	4 (2.9%)
Heartrate, bpm, median (IQR)	89 (77.75–118.50)	
Systolic blood pressure, mmHg, median (IQR)	142 (120–154)	
Diastolic blood pressure, mmHg, median (IQR)	80 (67–91)	
Laboratory values at admission		
Leucocytes (×10^9^), median (IQR)	10 (7.05–13.90)	6 (4.4%)
Lymphocytes (×10^9^), median (IQR)	1 (0.70–1.60)	30 (22%)
C-reactive protein (mg/L), median (IQR)	44 (17.75–118.50)	
Neutrophil-Lymphocyte ratio, median (IQR)	7.51 (3.96–14)	

^1^ body temperature > 38 °C. Abbreviations: IQR: interquartile range, brpm: breaths per minute, SpO_2_: Peripheral capillary oxygen saturation, bpm: beats per minute.

**Table 2:** Comorbidities.

Comorbidities	
Chronic cardiovascular, *n* (%)	103 (75.7%)
Hypertension, *n* (%)	79 (58.0%)
Peripheral arterial disease, *n* (%)	12 (8.8%)
Coronary artery disease or MI, *n* (%)	33 (24.2.%)
Previous stroke or TIA, *n* (%)	24 (23.3%)
Other ^1^, *n* (%)	63 (61.2%)
Diabetes mellitus Type 2, *n* (%)	27 (19.8%)
Chronic respiratory, *n* (%)	69 (51%)
COPD, *n* (%)	25 (18.3%)
Asthma, *n* (%)	16 (11.7%)
Sleep apnea syndrome, *n* (%)	8 (5.8%)
Other chronic respiratory diseases, *n* (%)	14 (10.2%)
Chronic kidney disease, *n* (%)	50 (36.7%)
Active cancer, *n* (%)	9 (6.7%)
Depression, *n* (%)	11 (8%)
Dementia, *n* (%)	16 (11.7%)
Neurological disorders	27 (19.8%)
State of immunosuppression, *n* (%)	11 (8.0%)
Chronic hematologic disease, *n* (%)	13 (9.5%)
Gastrointestinal, *n* (%)	25 (18.3%)
Chronic liver disease, *n* (%)	11 (8%)
Rheumatological, *n* (%)	13 (9.5%)

^1^ including: arrhythmia, congestive heart failure, valvular cardiomyopathy, takotsubo cardiomyopathy, cor pulmonale. Abbreviations: MI: myocardial infarction, TIA: transient ischemic attack, COPD: chronic obstructive pulmonary disease.

**Table 3:** Outcomes per comorbidity.

	*n*	Severe Course, *n* (%)	ICU, *n* (%)	In-Hospital Death, *n* (%)	LOHS ^1^, Median (IQR)
Overall	136	25 (18.4)	19 (14)	9 (6.6)	6 (4–10)
No comorbidity	2	1 (50)	1 (50%)	0	3.5 (3.2–3.7)
Coronary artery disease/MI	36	10 (27.8)	7 (19.4)	5 (13.9)	8 (4–11.5)
Arrythmia	34	5 (14.7)	4 (11.8)	3 (8.8)	7 (3.5–11)
Peripheral vascular disease	12	2 (16.7)	1 (8.3)	1 (8.3)	6 (3–9)
Diabetes mellitus Type 2	27	3 (11.1)	3 (11.1)	1 (3.7)	6 (3–10)
COPD	25	6 (24)	6 (24)	1 (4)	6 (4–9)
Asthma	16	3 (18.8)	2 (12.5)	2 (12.5)	5 (3.2–6.7)
Chronic kidney disease	50	13 (26)	8 (16)	8 (16)	6.5 (4–13)
Stroke or TIA	24	4 (16.7)	3 (12.5)	2 (8.3)	8 (6–11.7)
Chronic liver disease	12	3 (25)	2 (16.7)	2 (16.7)	6 (4.2 –8.7)
Immunosuppression	11	4 (36.4)	4 (36.4)	1 (9.1)	9 (6.2–14.2)
Chronic hematological disease	13	3 (23.1)	1 (7.7)	2 (15.4)	6 (4.2–8.7)

^1^ Length of hospital stay of survivors (*n* = 127). Abbreviations: MI: myocardial infarction, ICU: intensive care unit, LOHS: length of hospital stay, TIA: transient ischemic attack, COPD—chronic obstructive pulmonary disease, IQR—interquartile range.

## Data Availability

The data presented in this study are available from the corresponding author upon reasonable request. The data are not publicly available due to restrictions in data privacy.

## Abbreviations

The following abbreviations are used in this manuscript:

ARI	Acute respiratory infections
Brpm	Breaths per minute
Bpm	Beats per minute
BMI	Body mass index
CHF	Congestive heart failure
CKD	Chronic kidney disease
CLD	Chronic liver disease
COPD	Chronic obstructive pulmonary disease
CRP	C-reactive protein
e-GFR	Estimated glomerular filtration rate
ICU	Intensive care unit
IQR	Interquartile range
PCR	Polymerase chain reaction
RSV	Respiratory syncytial virus
SD	Standard deviation
SpO_2_	peripheral capillary oxygen saturation
TIA	Transient ischemic attack
